# Supplementary material for: Heat adaptation of phage T7 under an extended genetic code
Source: Virus Evol. 2021 Dec 1;7(2):veab100. doi: 10.1093/ve/veab100 (PMC8923235; doi:10.1093/ve/veab100)
Supplement: veab100_Supp [file veab100_supp.zip › Sequencing_Analysis.zip!/Sequencing_Analysis/S1/evidence/JC_SIDE_2_49.html]

BRESEQ :: Evidence


| New junction evidence | | | | | | | | | | | |
| --- | --- | --- | --- | --- | --- | --- | --- | --- | --- | --- | --- |
|  | | seq id | position | reads (cov) | reads (cov) | score | skew | freq | annotation | gene | product |
| \* | ? | NC\_001604.1 | = 7972 | 5047 (1.200) | 75 (0.020) | 61/588 | 24.5 | 1.5% | intergenic (–/–) | *–/–* | –/– |
|  | ? | NC\_001604.1 | 8524 = | 4926 (1.200) |  |  |  |  | intergenic (–/–) | *–/–* | –/– |
| Rejected: Coverage evenness skew score above cutoff. | | | | | | | | | | | |
| Rejected: Frequency below/above cutoff threshold. | | | | | | | | | | | |

Only 100 of 5011 total aligned reads displayed.  

`GGTGTATAGCTTGCTTAGAGAAAGATGACCGTTATCCACACACCTGTAACAAAGGAGCTAACGATATGACCGAACGTGAACAAGAGATGATCATTAAGTTGATAGACAATAATGAAGGTCGCCCAGATGATTTGAATGGCTGCGGTATTCTCTGCTCCAATGTCCCTTGCCACCTCTGCCCCGCAAATAACGATCAAAAGATAACCTTAGGTGAAATCCGAGCGATGGACCCACGTAAACCACATCTGAATAAACCTGAGGTAACTCCTACAGATGACCAGCCTTCCGCTGAGACAATCGAAGGTGTCACTAAGCCTTCCCACTACATGCTGTTTGACGACATTGAGGCTATCGAAGTGATTGCTCGTTCAATGACCGTTGAGCAGTTCAAGGGATACTGCTTCGGTAACATCTTAAAGTACAGACTACGTGCTGGTAAGAAGTCAGAGTTAGCGTACTTAGAGAAAGACCTAGCGAAAGCAGACTTCTATAAAGAACTCTTTGAGAAACATAAGGATAAATGTTATGCATAACTTCAAGTCAACCCCACCTGCCGACAGCCTATCTGATGACTTCACATCTTGCTCAGAGTGGTGCCGAA  >  NC_001604.1/8224‑8824`  

                                                                                                                                                                                                                                                                                                            `|`                                                                                                                                                                                                                                                                                                              
`ggTGTATAGCTTGCTTAGAGAAAGATGACCGTTATCCACACACCTGTAACAAAGGAGCTAACGATATGACCGAACGTGAACAAGAGATGATCATTAAGTTGATAGACAATAATGAAGGTCGCCCAGATGATTTGAATAGCTGCGGTATTCTCTGCTCCAATGTCCCTTGCCACCTCTGCCCCGCAAATAACGATCAAAAGATAACCTTAGGTGAAATCCGAGCGATGGACCCACGTAAACCACATCTGAATAAACCTGAGGTAACTCCTACAGATGACCAGCCTTCCGCTGAGACAATCGa                                                                                                                                                                                                                                                                                                              >  1:290878/1‑301 (MQ=255)`  
`ggTGTATAGCTTGCTTAGAGAAAGATGACCGGTATCCACACACCTGTAACAAAGGAGCTAACGATATTACCGAACGTGAACAAGAGATGATCATTAAGTTGATAGACAATAATGAAGGTCGCCCAGATGATTTGAATGGCTGCGGTATACTCTGCTCCAATGTCCCTTGCCACCTCTGCCCAGCAAATAACGATCAAAAGATAACCTTAGGTGAAATCCGAGCGATGGACCCACGTAAACCACATCTGAATAAACCTGAGGTAACTCATACAGATGACCAGCCTTCCGCTGAGACAATCGa                                                                                                                                                                                                                                                                                                              >  1:442854/1‑301 (MQ=255)`  
 `gtgtATAGCTTGCTTAGAGAAAGATGACCGTTATCCACACACCTGTAACAAAGGAGCTAACGATATGACCGAACGTGAACAAGAGATGATCATTAAGTTGATAGACAATAATGAAGGTCGCCCAGATGATTTGAATGGCTGCGGTATTCTCTGCTCCAATGTCCCTTGCCACCTCTGCCCCGCAAATAACGATCAAAAGATAACCTTAGGTGAAATCCGAGCAATGGACCCACGTAAACCACATCTGAATAAACCTGAGGTAACTCTTACAGATGACCCGCCTTCCGCTGAGACAATGGaa                                                                                                                                                                                                                                                                                                             >  1:95078/1‑301 (MQ=255)`  
 `gtgtATAGCTTGCTTAGAGAAAGATGACCGTTATCCACACACCTGTAACAAAGGAGCTAACGATATGACCGAACGTGAACAAGAGATGATCATTAAGTTGATAGACAATAATGAAGGTCGCCCAGATGATTTGAATGGCTGCGGTATTCTCTGCTCCAATGTCCCCTGCCACCTCTGCCCCGCAAATAACGATCAAAAGATAACCTTAGGTGAAATCCGAGCGATGGACCCACGTAAACCACATCTGAATAAACCTGAGGTAACTCCTACAGATGACCAGCCTTCCGCTGAGACAATCGaa                                                                                                                                                                                                                                                                                                             >  1:357927/1‑301 (MQ=255)`  
       `aGCTTGCTTAGAGAAAGATGACCGTTATCCACACACCTGTAACAAAGGAGCTAACGATATGACCGAACGTGAACAAGAGATGATCATTAAGTTGATAGACAATAATGAAGGTCGCCCAGATGATTTGAATGGCTGCGGTATTCTCTGCTCCAATGTCCCTTGCCACCTCTGCCCCGCAAATAACGATCAAAAGATAACCTTAGGTGAAATCCGAGCGATGGACCCACGTAAACCACATCTGAATAAACCTGAGGTAACTCCTACAGATGACCAGCCTTCCGCTGAGACAATCGAAGgtgtt                                                                                                                                                                                                                                                                                                       >  1:216754/1‑300 (MQ=255)`  
             `cttAGAGAAAGATGACCGTTATCCACACACCTGTAACAAAGGAGCTAACGATATGACCGAACGTGAACAAGAGATGATCATTAAGTTGATAGACAATAATGAAGGTCGCCCAGATGATTTGAATGGCTGCGGTATTCTCTGCTCCAATGTCCCTTGCCACCTCTGCCCCGCAAATAACGATCAAAAGATAACCTTAGGTGAAATCCGAGCGATGGACCCACGTAAACCACATCTGAATAAACCTGAGGTAACTCCTACAGATGACCAGCCTTCCGCTGAGACAATCGAAGGTGTCACTAAg                                                                                                                                                                                                                                                                                                 <  1:153308/301‑1 (MQ=255)`  
              `gtaaatgtttataagtgacagccACACACCTGTAACAACTGCGCTAACGTTATGACCGAACGTGAACAAGTGATGATCATTCAGTTGATAGACAATAATGAATATCACCCAGATGATTAGAATGGCTGCAGTATTCTCTGCTCCAATGTCCCTTGCCACCTATGCCCCGCAAATAACGATCAAAAGATAACCTTAGGTGAAATCCGAGCAATGGACCCACATAAACCACATCTGAATAAACCTGAGGTAACTCCTACAGATGACCAACCTTCCGCTGAGACAATCGAAGGTGTCACTAAGc                                                                                                                                                                                                                                                                                                <  1:585809/280‑1 (MQ=255)`  
               `tAGAGAAAGATGACCGTTATCCACACACCTGTAACAAAGGAGCTAACGATATGACCGAACGTGAACAAGAGATGATCATTAAGTTGATAGACAATAATGAAGGTCGCCCAGATGATTTGAATGGCTGCGGTATTCTCTGCTCCAATGTCCCTTGCCACCTCTGCCCCGCAAATAACGATCAAAAGATAACCTTAGGTGAAATCCGAGCGATGGACCCACGTAAACCACATCTGAATAAACCTGAGGTAACTCCTACAGATGACCAGCCTTCCGCTGAGACAATCGAAGGTGTCACTAAGcc                                                                                                                                                                                                                                                                                               <  1:592174/301‑1 (MQ=255)`  
               `gtgtataagagacaggTTATCCACACACCTGTAACAAAGGAGCTAACGATATGACCGAACGTGAACAAGAGATGATCATTAAGTTGATAGACAATAATGAAGGTCGCCCAGATGATTTGAATGGCTGCGGTATTCTCTGCTCCAATGTCCCTTGCCACCTCTGCCCCGCAAATAACGATCAAAAGATAACCTTAGGTGAAATCCGAGCGATGGACCCACGTAAACCACATCTGAATAAACCTGAGGTAACTCCTACAGATGACCAGCCTTCCGCTGAGACAATCGAAGGTGTCACTAAGcc                                                                                                                                                                                                                                                                                               <  1:398613/286‑1 (MQ=255)`  
                  `agaAAGATGACCGTTATCCACACACCTGTAACAAAGGAGCTAACGATATGACCGAACGGTAACAAGAGATGATCATTAAGTTGATAGACAATAATGAAGGTCGCCCAGATGATTTGAATGGCTGCGGTATTCTCTGCTCCAATGTCCCTTGCCACCTCTGCCCCGCAAATAACGATCAAAAGATAACCTTAGGTGAAATCCGAGCGATGGACCCACGTAAACCACATCTGAATAAACCTGAGGTAACTCCTACAGATGACCAGCCTTCCGCTGAGACAATCGAAGGTGTCACTAAGCCTTc                                                                                                                                                                                                                                                                                            <  1:598020/301‑1 (MQ=255)`  
                   `gaAAGATGACCGTTATCCACACACCTGTAACAAAGGAGCTAACGATATTACCGAACGTGAACAAGAGATGATCATTAAGTTGATAGACAATAATGAAGGTCGCCCAGATGATTTGAATGGCTGCGGTATTCTCTGCTCCAATGTCCCTTGCCACCTCTGCCCCGCAAATAACTATCAAAAGATAACCTTAGGTGAAATCCGAGCGATGGACCCACGTAAACCACATCTGAATAAACCTGAGGTAACTCCTACAGATGACCAGCCTTCCGCTGAGACAATCGAAGGTGTCACTAAGccctgt                                                                                                                                                                                                                                                                                           >  1:88659/1‑297 (MQ=255)`  
                      `tgtgtataagagacagcacacCTGTAACAAAGGAGCTAACGATATGACCGAACGTGAACAAGAGATGATCATTAAGTTGATAGACAATAATGAAGGTCGCCCAGATGATTTGAATGGCTGCGGTATTCTCTGCTCCAATGTCCCTTGCCACCTCTGCCCCGCAAATAACGATCAAAAGATAACCTTAGGTGAAATCCGAGCGATGGACCCACGTAAACCACATCTGAATAAACCTGAGGTAACTCCTACAGATGACCAGCCTTCCGCTGAGACAATCGAAGGTGTCACTAAGCCTTCCCAc                                                                                                                                                                                                                                                                                        <  1:547700/285‑1 (MQ=255)`  
                      `aGATGACCGTTATCCACACACCTGTAACAAAGGAGCTAACGATATGACCGAACGTGAACAAGAGATGATCATTAAGTTGATAGACAATAATGAAGGTCGCCCAGATGATTTGAATGGCTGCGGTATTCTCTGCTCCAATGTCCCTTGCCACCTCTGCCCCGCAAATAACGATCAAAAGATAACCTTAGGTGAAATCCGAGCGATGGACCCACGTAAACCACATCTGAATAAACCTGAGGTAACTCCTACAGATGACCAGCCTTCCGCTGAGACAATCGAAGGTGTCACTAAGCCTTCCCAc                                                                                                                                                                                                                                                                                        >  1:640164/1‑301 (MQ=255)`  
                         `ggACCGTTATCCACACACCTGTAACAAAGGAGCTAACGATATGACCGAACGTGAACAAGAGATGATCATTAAGTTGATAGACAATAATGAAGGTCGCCCAGATGATTTGAATGGCTGCGGTATTCTCTGCTCCAATGTCCCTTGCCACCTCTGCCCCGCAAATAACGATCAAAAGATAACCTTAGGTGAAATCCGAGCGATGGACCCACGTAAACCACATCTGAATAAACCTGAGGTAACTCCTACAGATGACCAGCCTTCCGCTGAGACAATCGAAGGTGTCACTAAGCCTTCCCACTAc                                                                                                                                                                                                                                                                                     <  1:337552/300‑1 (MQ=255)`  
                           `aCCGTTATCCACACACCTGTAACAAAGGAGCTAACGATATGACCGAACGTGAACAAGAGATGATCATTAAGTTGATAGACAATAATGAAGGTCGCCCAGATGATTTGAATGGCTGCGGTATTCTCTGCTCCAATGTCCCTTGCCACCTCTGCCCCGCAAATAACGATCAAAAGATAACCTTAGGTGAAATCCGAGCGATGGACCCACGTAAACCACATCTGAATAAACCTGAGGTAACTCCTACAGATGACCAGCCTTCCGCTGAGACAATCGAAGGTGTCACTAAGCCTTCCCACTACAt                                                                                                                                                                                                                                                                                   >  1:553084/1‑301 (MQ=255)`  
                           `aCCGTTATCCACACACCTGTAACAAAGGAGCTAACGATATGACCGAACGTGAACAAGAGATGATCATTAAGTTGATAGACAATAATGAAGGTCGCCCAGATGATTTAAATGGCTGCGGTATTCTCTGCTCCAATGTCCCTTGCCACCTCTGCCCCGCAAATAACGATCAAAAGATAACCTTAGGTGAAATCCGAGCGATGGACCCTCGTAAACCACATCTGAATAAACCTGAGGTAACTCCTACAGATGACCAGCCTTCCGCTGAGACAATCGAAGGTGTCACTAAGCCTTCCCACTACAt                                                                                                                                                                                                                                                                                   <  1:277618/301‑1 (MQ=255)`  
                              `gTTATCCACACAACTGTAACAAAGGAGCTAACGATATGACCGAACGTGAACAAGAGATGATCATTAAGTTGATAGACAATAATGAAGGTCGCCCAGATGATTTGAATGGCTGCGGTATTCTCTGCTCCAATGTCCCTTGCCACCTCTGCCCCGCAAATAACGATCAAAAGATAACCTTAGGTGAAATCCGAGCGATGGACCCACGTAAACCACATCTGAATAAACCTGAGGTAACTCCTACAGATGACCAGCCTTCCGCTGAGACAATCGAAGGTGTCACTAAGCCTTCCCACTACATGCt                                                                                                                                                                                                                                                                                <  1:12480/301‑1 (MQ=255)`  
                                 `aagagaaagccTGTAACAAAGGAGCTAACGATATGACCGAACGTGAACAAGAGATGATCATTAAGTTGATAGACAATAATGAAGGTCGCCCAGATGATTTGAATGGCTGCGGTATTCTCTGCTCCAATGTCCCTTGCCACCTCTGCCCCGCAAATAACGATCAAAAGATAACCTTAGGTGAAATCCGAGCGATGGACCCACGTAAACCACATCTGAATAAACCTGAGGTAACTCCTACAGATGACCAGCCTTCCGCTGAGACAATCGAAGGTGTCACTAAGCCTTCCCACTACATGCTGtt                                                                                                                                                                                                                                                                             <  1:578828/292‑1 (MQ=255)`  
                                   `ccACACACCTGTAACAAAGGAGCTAACGATATGACCGAACGTGAACAAGAGATGATCATTAAGTTGATAGACAATAATGAAGGTCGCCCAGATGATTTGAATGGCTGCGGTATTCTCTGCTCCAATGTCCCTTGCCACCTCTGCCCCGCAAATAACGATCAAAAGATAACCTTAGGTGAAATCCGAGCGATGGACCCACGTAAACCACATCTGAATAAACCTGAGGTAACTCCTACAGATGACCAGCCTTCCGCTGAGACAATCGAAGGTGTCACTAAGCCTTCCCACTACATGCTGTTTg                                                                                                                                                                                                                                                                           >  1:430593/1‑301 (MQ=255)`  
                                          `ccTGTAACAAAGGAGCTAACGATATGACCGAACGTGAACAAGAGATGATCATTAAGTTGATAGACAATAATGAAGGTCGCCCAGATGATTTGAATGGCTGCGGTATTCTCTGCTCCAATGTCCCTTGCCACCTCTGCCCCGCAAATAACGATCAAAAGATAACCTTAGGTGAAATCCGAGCGATGGACCCACGTAAACCACATCTGAATAAACCTGAGGTAACTCCTACAGATGACCAGCCTTCCGCTGAGACAATCGAAGGTGTCACTAAGCCTTCCCACTACATGCTGTTTGACGACAt                                                                                                                                                                                                                                                                    >  1:653204/1‑301 (MQ=255)`  
                                             `gTAACAAAGGAGCTAACGATATGACCGAACGTGAACAAGAGATGATCATTAAGTTGATAGACAATAATGAAGGTCGCCCAGATGATTTGAATGGCTGCGGTATTCTCTGCTCCAATGTCCCTTGCCACCTCTGCCCCGCAAATAACGATCAAAAGATAACCTTAGGTGAAATCCGAGCGATGGACCCACGTCAACAACATCTGAATAAACCTGAGGTAACTCCTACAGATGACCAGCCTTCCGCTGAGACAATCGAAGGTGTCACTAAGCCTTCCCACTACATGCTCtttctcttatacac                                                                                                                                                                                                                                                                 >  1:374782/1‑290 (MQ=255)`  
                                              `tAACAAAGGAGCTAACGATATGACCGAACGTGAACAAGAGATGATCATTAAGTTGATAGACAATAATGAAGGTCGCCCAGATGATTTGAATGGCTGCGGTATTCTCTGCTCCAATGTCCCTTGCCACCTCTGCCCCGCAAATAACGATCAAAAGATAACCTTAGGTGAAATCCGAGCGATGGACCCACGTAAACCACATCTGAATAAACCTGAGGTAACTCCTACAGATGACCAGCCTTCCGCTGAGACAATCGAAGGTGTCACTAAGCCTTCCCACTACATGCTGTTTGACGACATTGAg                                                                                                                                                                                                                                                                >  1:297381/1‑301 (MQ=255)`  
                                                 `cAAAGGAGCTAACGATATGACCGAACGTGAACAAGAGATGATCATTAAGTTGATAGACAATAATGAAGGTCGCCCAGATGATTTGAATGGCTGCGGTATTCTCTGCTCCAATGTCCCTTGCCACCTCTGCCCCGCAAATAACGATCAAAAGATAACCTTAGGTGAAATCCGAGCGATGGACCCACGTAAACCACATCTGAATAAACCTGAGGTAACTCCTACAGATTACCAGCCTTCCGCTGAGACAATCGAAGGTGTCACTAAGCCTTCCCACTACATGCTGTTTGACGACATTGAGGCt                                                                                                                                                                                                                                                             >  1:149038/1‑301 (MQ=255)`  
                                                     `ggAGCTAACTATATGACCGAACGTGAACAAGAGATGATCATTAAGTTGATAGACAATAATGAAGGTCGCCCAGATGATTTGAATGGCTGCGGTATTCTCTGCTCCAATGTCCCTTGCAACCTCTGCCCCGCAAATAACGATCAAAAGATAACCTTAGGTGAAATCCGAGCGATGGACCCACGTAAACCACATCTGAATAAACCTGAGGTAACTCCTACAGATGACCAGCCTTCCGCTGAGACAATCGAAGGTGTCACTAAGCCTTCCCACTACATGCTGTTTGACGACATTGAGGcctgtc                                                                                                                                                                                                                                                         >  1:189479/1‑296 (MQ=255)`  
                                                          `aaaCGATATGACAGAACGTGAACAAGAGATGATCATTAAGTTGATAGACAATAATGAAGGTCGCCCAGATGATTTGAATGGCTGCGGTATTCTCTGCTCCAATGTCCCTTGCCACCTCTGCCCCGCAAATAACGATCAAAAGATAACCTTAGGTGAAATCCGAGCGATGGACCCACGTAAACCACATCTGAATAAACCTGAGGTAACTCCTACAGATGACCAGCCTTCCGCTGAGACAATCGAAGGTGTCACTAAGCCTTCCCACTACATGCTGTTTGACGACATTGAGGCTATCGAAGTg                                                                                                                                                                                                                                                    <  1:129604/300‑1 (MQ=255)`  
                                                               `atatGACCGAACGTGAACAAGAGATGATCATTAAGTTGATAGACAATAATGAAGGTCGCCCAGATGATTTGAATGGCTGCGGTATTCTCTGCTCCAATGTCCCTTGCCACCTCTGCCCCGAAAATAACGATCAAAAGATAACCTTAGGTGAAATCCGAGCGATGGACCCACGTAAACCACATCTGAATAAACCTGAGGTAACTCCTACAGATGACCAGCCTTCCGCTGAGACAATCGAAGGTGTCACTAAGCCTTCCCACTACATGCTGTTTGACGACATTGAGGCTATCGAAGTGATTGc                                                                                                                                                                                                                                               >  1:100098/1‑301 (MQ=255)`  
                                                               `acaggACCGAACTTGAACAAGCGATGATCATAAAGTTGATAGACAATAATGAAGGTCGCCCAGATGAGTTTAATGTCTGCGGTATTCTCTGCTCCACTGTCCCTTGCCACCTCTGCCCCGCAAATAACGATCAAAAGGTAACCTTAGGTGAAATCCGAGCGATGGACCCACGTAAACTACCTCTGAATACACCTGAGGTAACTCCTACAGTTGACCAGCCTTCCGCTGATACAATCGAAGGTTTCACTAAGCCTTCCCACTACATGCTGTTTGACGACATTGAGGCTATCGAAGTGATTGc                                                                                                                                                                                                                                               <  1:134755/297‑1 (MQ=255)`  
                                                                `ggctcggagatgtgtataagcgacagtCATTAAGTTGATAGGCAATAATGAAGGTCTCCCAGATGATTTGAATGGCTGCGGTATTCTCTGCTCCAATGTCCCTTGCCACCTCTGCCCCGCAAATAACGATCAAAAGATAACCTTAGGTGAAATCCGAGCGATGGACCCACGTAAACCACATCTTAATCAACCTGAGGTAACTCCTACAGATGACCAGCCTTCCGCTGAGACAATCGAAGGTGTCACTAAGCCTTGCCACTACATGCTGTTTGACGACATTGAGGCTATCGAAGTGATTGCt                                                                                                                                                                                                                                              <  1:610868/275‑1 (MQ=255)`  
                                                                        `aaCGTGAACAAGAGATGATCATTAAGTTGATAGACAATAATGAAGGTCGCCCAGATGATTTGAATGGCCGCGGTATTCTCTGCTCCAATGTCCCTTGCCACCTCTGCCCCGCAAATAACGATCAAAAGATAACCTTAGGTGAAATCCGAGCGATGAACCCACGTAAACCACATCTGAATAAACCTGAGGTAACTCCTACAGATGACCAGCCTTCCGCTGAGACAATCGAAGGTGTCACTAAGCCTTCCCACTACATGCTGTTTGACGACATTGAGGCTATCGAAGTGTTTGCTCGTTCAAt                                                                                                                                                                                                                                      >  1:27101/1‑301 (MQ=255)`  
                                                                                  `agagATGATCATTAAGTTGATAGACAATAATGAAGGTCGCCCAGATGATTTGAATGGCTGCGGTATTCTCTGCTCCAATGTCCCTTGCCACCTCTGCCCCGCAAATAACGATCAAAAGATAACCTTAGGTGAAATCCGAGCGATGGACCCACGTAAACCACATCTGAATAAACCTGAGGTAACTCCTACAGATGACCAGCCTTCCGCTGAGACAATCGAAGGTGTCACTAAGCCTTCCCACTACATGCTGTTTGACGACATTGAGGCTATCGAAGTGATTGCtgtctcttatacacatctc                                                                                                                                                                                                                            >  1:411444/1‑283 (MQ=255)`  
                                                                                             `ttAAGTTGATAGACATTAATGAAGGTCGCCCAGATGATTTGAATGGCTGCGGTATTCTCTGCTCCAATGTCCCTTGCCACCTCTGCCCCGCAAATAACGATCAAAAGATAACCTTAGGTGAAATCCGAGCGATGGACCCACGTAAACCACATCTGAATAAACCTGAGGTAACTCCTACAGATGACCAGCCTTCCGCTGAGACAATCGAAGGTGTCACTAAGCCTTCCCACTACATGCTGTTTGACGACATTGAGGCTATCGAAGTGATTGCTCGTTCAATGACCGTTGAGCAGTTCAAggg                                                                                                                                                                                                                 <  1:418306/301‑1 (MQ=255)`  
                                                                                             `gtgtataagagacagataatGAAGGTCGCCCAGATGATTTGAATGGCTGCGGTATTCTCTGCTCCAATGTCCCTTGCCACCTCTGCCCCGCAAATAACGATCAAAAGAGAACCTTAGGTGAAATCCGAGCGATGGACCCACGTAAACCACATCTGAATAAACCTGAGGTAACTCCTACAGATGACCAGCCTTCCGATGAGACAATCGAAGGTGTCACTAAGCCTTCCCACTACATGCTGTTTGACGACATTGAGGCTATCGAAGTGATTGCTCGTTCAATGACCGTTGAGCAGTTCAAggg                                                                                                                                                                                                                 <  1:634474/286‑1 (MQ=255)`  
                                                                                                `aGTTGATAGACACTAATGAAGGTCGCCCAGATGATTTGAATGGCTGCGGTATTCTCTGCTCCAATGTCCCTTGCCACCTCTGCCCCGCAAATAACGATCAAAAGATAACCTTAGGTGAAATCCGAGCGATGGACCCACGTAAACCACATCTGAATAAACCTGAGGTAACTCCTACAGATGACCAGCCTTCCGCTGAGACAATCGAAGGTGTCACTAAGCCTTCCCACTACATGCTGTTTGACGACATTGAGGCTATCGAAGTGATTGCTCGTTCAATGACCGTTGAGCAGTTAAAGGGATa                                                                                                                                                                                                              >  1:313856/1‑301 (MQ=255)`  
                                                                                                `aGTTGATAGACAATAATGAAGGTCGCCCAGATGATTTGAATGGCTGCGGTATTCTCTGCTCCAATGTCCCTTGCCACCTCTGCCCCGCAAATAACGATCAAAAGATAACCTTAGGTGAAATCCGAGCGATGGACCCACGTAAACCACATCTGAATAAACCTGAGGTAACTCCTACAGATGACCAGCCTTCCGCTGAGACAATCGAAGGTGTCACTAAGCCTTCCCACTACATGCTGTTTGACGACATTGAGGCTATCGAAGTGATTGCTCGTTCAATGACCGTTGAGCAGTTCAAGGGATa                                                                                                                                                                                                              >  1:205621/1‑301 (MQ=255)`  
                                                                                                  `ttGATAGACAATAATGAAGGTCGCCCAGATGATTTGAATGGCTGCGGTATTCTCTGCTCCAATGTCCCTTGCCACCTCTGCCCCGCAAATAACGATCAAAAGATAACCTTAGGTGAAATCCGAGCGATGGACCCACGTAAACCACATCTGAATCAACCTGAGGTAACTCCTACAGACGACCAGCCTTCCGCTGAGACAATCGAAGGTGTCACTAAGCCTTCCCACTACATGCTGTTTGACGACATTGAGGCTATCGAAGTGATTGCTCGTTCAATGACCGTTGAGCAgctgtctcttatac                                                                                                                                                                                                            >  1:23450/1‑288 (MQ=255)`  
                                                                                                    `taTAGACAATAAGGAAGGTCGCCCATATGATTTGAATGGCTGCGGTATTCTCTGCGCCAATGTCCCTTGCCACCTCTGCCCCGCAAATAACGATCAAAAGATAACCTTAGGTGAAATCCGAGCGATGGACCCACGTAAACCACATCTGAATAAACCTGAGGTAACTCCTACAGATGACCAGCCTTCCGCTGAGACAATCGAAGGTGTCACTAAGCCTTCCCACTACATGCTGTTTGACGACATTGAGGCTATCGAAGTGATTGCTCGTTCAATGACCGTTGAGCAGTTCAAGGGATACTGc                                                                                                                                                                                                          <  1:669124/300‑1 (MQ=255)`  
                                                                                                          `cggagatgtgtataagagacaggatTTGAATGGCTGCGGTATTCTCTGCTCCAATGTCCCTTGCCACCTCTGCCCCGCAAATAACGATCAACAGATAACCTTAGGTGAAATCCGAGCGATGGACCCACGTAAACCACATCTGAATAAACCTGAGGTAACTCCTACAGATGACCAGCCTTCCGCTGAGACAATCGAAGGTGTCACTAAGCCTTCCCACTACATGCTGTTTGACGACATTGAGGCTATCGAAGTGATTGCTCGTTCAATGACCGTTGAGCAGTTCAAGGGATACTGCTTCGGt                                                                                                                                                                                                    <  1:483088/279‑1 (MQ=255)`  
                                                                                                          `cAATAATGAAGGTCGCCCAGATGATTTGAATGGCTGCGGTATTCTCTGCTCCAATGTCCCTTGCCACCTCTGCCCCGCAAATAACGATCAACAGATAACCTTAGGTGAAATCCGAGCGATGGACCCACGTAAACCACATCTGAATAAACCTGAGGTAACTCCTACAGATGACCAGCCTTCCGCTGAGACAATCGAAGGTGTCACTAAGCCTTCCCACTACATGCTGTTTGACGACATTGAGGCTATCGAAGTGATTGCTCGTTCAATGACCGTTGAGCAGTTCAAGGGCTACTGCTTCGGt                                                                                                                                                                                                    >  1:47613/1‑301 (MQ=255)`  
                                                                                                          `cAATAATGAAGGTCGCCCAGATGATTTGAATGGCTGCGGTATTCTCTGCTCCAATGTCCCTTGCCACCTCTGCCCCGCAAATAACGATCAAAAGATAACCTTAGGTGAAATCCGAGCGATGGACCCACGTAAACCACATCTGAATAAACCTGAGGTAACTCCTACAGATGACCAGCCTTCCGCTGAGACAATCGAAGGTGTCACTAAGCCTTCCCACTACATGCTGTTTGACGACATTGAGGCTATCGAAGTGATTGCTCGTTCAATGACCGTTGAGCAGTTCAAGGGATACTGCTTCGGt                                                                                                                                                                                                    <  1:264562/301‑1 (MQ=255)`  
                                                                                                          `cAATAATGAAGGTCGCCCAGATGATTTGAATGGCTGCGGTATTCTCTGCTCCAATGTCCCTTGCCACCTCTGCCCCACAAATAACGATCAAAAGATAACCTTAGGTGAAATCCGAGCGATGGACCCACGTAAACCACATCTGAATAAACCTGAGGTAACTCCTACAGATGACCAGCCTTCCGCTGAGACAATCGAAGGTGTCACTAAGCCTTCCCACTACATGCTGTTTGACGACATTGAGGCTATCGAAGTGATTGCTCGTTCATTGACCGTTGAGCAGTTCAAggctgtctcttatacc                                                                                                                                                                                                    >  1:424332/1‑287 (MQ=255)`  
                                                                                                            `ataatGAAGGTCGTCCAGATGATTTGAATGGCTGCGGTATTCTCTGCTCCAATGTCCCTTGCCACCTCTGCCCCGCAAATAACGATCAAAAGATAACCTTAGGTGAAATCCGAGCGATGGACCCACGTAAACCACATCTGAATAAACCTGAGGTAACTCCTACAGATGACCAGCCTTCAGCTGAGACAATCGAAGGTGTCACTAAGCCATCCCACTACATGCTGTTTGACGACATTGAGGCTATCGAAGTGATTGCTCGTTCAATGACCGTTGAGCAGTTCAAGGGAGACTGCATCGGTaa                                                                                                                                                                                                  >  1:500651/1‑301 (MQ=255)`  
                                                                                                            `ataatGAAGGTCGCCCAGATGATTTGAATGGCTGCGGTATTCTCTGCTCCAATGTCCCTTGCCACCTCTGCCCCGCAAATAACGATCAAAAGATAACCTTAGGTGAAATCCGAGCGATGGACCCACGTAAACCACATCTGAATAAACCTGAGGTAACTCCTACAGATGACCAGCCTTCCGCTGAGACAATCGAAGGTGTCACTAAGCCTTCCCACTACATGCTGTTTGACGACATTGAGGCTATCGAAGTGATTGCTCGTTCAATGAACGTTGAGCAGTTCAAgggctgtctcttatacac                                                                                                                                                                                                  >  1:259896/1‑286 (MQ=255)`  
                                                                                                                `tGAAGGTCGCCCAGAGGATTTGAATGGATGCGGTATTCTCTGCTCCAATGTCCCTTGCCACCTCTGCCCCGCAAATAACGATCAAAAGATAACCTTAGGTGAAATCCGAGCGATGGACCCACGTAAACCACATCTGAATAAACCTGAGGTAACTCCTACAGATGACCAGCCTTCCGCTGAGACAATCGAAGGTGTCACTAAGCCTTCCCACTACATGCTGTTTGACGACATTGAGGCTATCGAAGTGATTGCTCGTTCAATGACCGTTGAGCAGTTCAAGGGATACTGCTTCGGTAACATc                                                                                                                                                                                              <  1:228115/301‑1 (MQ=255)`  
                                                                                                                `agAAGGTCGCCCAGATGATTTGAATGGCTGCGGTAGTCTCTTCTCCAATGTCCCTTGCCACCTCTGCCCCGCAAATAACGATCAAAAGATAAACTTAGGTGAAATCCGAGCGATGGACCCACGTAAACCACATCTGAATAAACCTGAGGTAACTCCTACAGATGACCAGCCTTCCGCTGAGACAATCGAAGGTGTCACTAAGCCTTCCCACTACATGCTGTTTGACGACATTGAGGCTATCGAAGTGATTGCTCGTTCAATGACCGTTGAGCAGTTCAAGGGATACTGCTTCGGTAACATc                                                                                                                                                                                              <  1:393114/300‑1 (MQ=255)`  
                                                                                                                 `aaaGGTCGCCCAGAAGATTTGAAAGGCTGCTGTATTCTATGCTCCAATGTACATTGCCACATCTGACCCGCAAATAACGATCAAAAGATAACATTAGGTGAAATCCGAGCGATGGACCCACGTAAACCACAACTGAATAAACCTGAGGTAACTCCTACAGATGACCAGCCTTCCGCTGAGACAATCGAAGGTGTCACTAAGCCTTCCCACTACATGCTGTTTGACGACATTGAGGCTATCGAAGTGATTGCTCGTTAAATGACCGTTGAGCAGTTCAAGGGATACTGCTTCGGTAACATCt                                                                                                                                                                                             <  1:46210/300‑1 (MQ=255)`  
                                                                                                                       `cGCCCAGATGATTTGAATGGCTGCGGTATTCTCTGCTCCAATGTCCCTTGCCACCTCTGCCCCGCAAATAACGATCAAAAGATAACCTTAGGTGAAATCCGAGCGATGGACCCACGTAAACCACATCTGAATAAACCTGAGGTAACTCCTACAGATGACCAGCCTTCCGCTGAGACAATCGAAGGTGTCACTAAGCCTTCCCACTACATGCTGTTTGACGACATTGAGGCTATCGAAGTGATTGCTCGTTCAATGACCGTTGAGCAGTTCAAGGGATACTGCTTCGGTAACATCTTAAAGt                                                                                                                                                                                       <  1:171036/301‑1 (MQ=255)`  
                                                                                                                             `gatgatTTGAATGGCTGCGGTATTCTCTGCTCCAATGTCCCTTGCCACCTCTGCCCCGCAAATAACGATCAAAAGATAACCTTAGGTGAAATCCGAGCGATAGACCCACGTAAACCACATCTGAATAAACCTGAGGTAACTCCTACAGATGACCAGCCTTCCTCTGAGACAATCGAAGGTGTCACTAAGCCTTCCCACTACATGCTGTTTGACGACATTGAGGCTATCGAAGTGATTGCTCGTTCAATGACCGTTGAGCAGTTCAAGGGATACTGCTTCGGTAACATCTTAAAGTACAGAc                                                                                                                                                                                 <  1:177994/301‑1 (MQ=255)`  
                                                                                                                              `tgtgtataagagacagCGGTATTCTCTGCTCCAATGTCCCTTGCCACCTCTGCCCCGCAAATAACGATCAAAAGATAACCTTAGGTGAAATCCGAGCGATGGACCCACGTAAACCACATCTGAATAAACCTGAGGTAACTCCTACAGATGACCAGCCTTCCGCTGAGACAATCGAAGGTGTCACTAAGCCTTCCCACTACATGCTGTTTGACGACATTGAGGCTATCGAAGTGTTTGCTCGTTCAATGACCGTTGAGCTGTTCAAGGGATACTGCTTCGGTAACATCTTAAAGTACAGACt                                                                                                                                                                                <  1:559703/286‑1 (MQ=255)`  
                                                                                                                               `ggatTTGAATGGCTGCGGTATTCTCTGCTCCAATGTCCCTTGCCACCTCTGCCCCGCAAATAACGATCAAAAGATAACCTTAGGTGAAATCCGAGCGATGGACCCACGTAAACCACATCTGAATAAACCTGAGGTAACTCCTACAGATGACCAGCCTGCCGCTGAGACAATCGAAGGTGTCACTAAGCCTTCCCACTACATGCTGTTTGACGACATTGAGGCTATCGAAGTGATTGCTCGTTCAATGACCGTTGAGCAGTTCAAGGGATACTGCTTCGGTAACATCTTAAAGTACAGACTa                                                                                                                                                                               <  1:194987/300‑1 (MQ=255)`  
                                                                                                                                `gatTTGAATGGCTGCGGTATTCTCTGCTCCAATGTCCCTTGCCACCTCTGCCCCGCAAATAACGATCAAAAGATAACCTTAGGTGAAATCCGAGCGATGGACCCACGTAAACCACATCTGAATAAACCTGAGGTAACTCCTACAGATGACCAGCCTTCCGCTGAGACAATCGAAGGTGTCACTAAGCCTTCCCACTACATGCTGTTTGACGACATTGAGGCTATCGAAGTGATTGCTCGTTCAATGACCGTTGAGCAGTTCAAGGGATACTGcctgtctcttatacacatctccgagcccc                                                                                                                                                                              >  1:604811/1‑273 (MQ=255)`  
                                                                                                                                   `ttaaaTGGCGGCGATATGCGCTGCTCCTATGTCCCTAGCCACCTCGGCCCCGCAAATAACGAACAGAAGATAACCGTAGGTGATATCCGAGCTATGAACCCACGGAAACCACATGTGAATGAAACTTAGGTAGCTCCTACAGATGACCAGCCGTCCGCTGGGACAAGCGAAGGTGTCACTAAACCTTCCCACTACTGACTGTGTGACGACATTGAGGCTATCGAAGTGATTGCTCGTTCAATGGCCGTTAAGCAGTTCAAGGTATACTGCTTCGGTAACATCTTAAAGTACAGACTACGTg                                                                                                                                                                           <  1:487750/298‑1 (MQ=255)`  
                                                                                                                                   `ttGAATGGCTGCGGTATTCTCTGCTCCAATGTCCCTTGCCACCTCTGCCCCGCAAATAACGATCAAAAGATAACCTTAGGTGAAATCCGAGCGATGGACCCACGTAAACCACATCTGAATAAACCTGAGGTAACTCCTACAGATGACCAGCCTTCCGCTGAGACAATCGAAGGTGTCACTAAGCCTTCCCACTACATGCTGTTTGACGACATTGAGGCTATCGAAGTGATTGCTCGTTCAATGACCGTTGAGCAGTTCAAGGGATACTGCTTCGGTAACATCTTAAAGTACAGACTACGTg                                                                                                                                                                           <  1:246617/301‑1 (MQ=255)`  
                                                                                                                                    `tGAATGGCTGCGGTATTCTCTGCTCCAATGTCCCTTGCCACCTCTGCCCCGCAAATAACGATCAAAAGATAACCTTAGGTGAAATCCGAGCGATGGACCCACGTAAACCACATCTGAATAAACCTGAGGTAACTCCTACAGATGACCAGCCTTCCGCTGAGACAATCGAAGGTGTCACTAAGCCTTCCCACTACATGCTGTTTGACGACATTGAGGCTATCGAAGTGATTGCTCGTTCAATGACCGTTGAGCAGTTCAAGGGATACTGCTTCGGTAACATCTTAAAGTACAGACTACGTGc                                                                                                                                                                          <  1:76265/301‑1 (MQ=255)`  
                                                                                                                                      `aaTGGCTGCGGTATTCTCTGCTCCAATGTCCCTTGCCACCTCTGCCCCGCAAATAACGATCAAAAGATAACCTTAGGTGAAATCCGAGCGATGGACCCACGTAAACCACATCTGAATAAACCTGAGGTAACTCCTACAGATGACCAGCCTTCCGCTGAGACAATCGAAGGTGTCACTAAGCCTTCCCACTACATGCTGTTTGACGACATTGAGGCTATCGAAGTGATTGCTCGTTCAATGACCGTTGAGCAGTTCAAGGGATACTGCTTCGGTAACATCTTAAAGTACAGACTACATGCTg                                                                                                                                                                        <  1:210240/301‑1 (MQ=255)`  
                                                                                                                                             `gCGGTATTCTCTGCTCCAATGTCCCTTGCCACCTCTGCCCCGCAAATAACGATCAAAAGATAACCTTAGGTGAAATCCGAGCGTTGGACCCACGTAAACCACATCTGAATAAACCTGAGGTAACTCCTACAGATGACCAGCCTTCCGCTGAGACAATCGAAGGTGTCACTAAGCCTTCCCACTACATGCTGTTTGACGACATTGAGGCTATCGAAGTGATTGCTCGTTCAATGACCGTTGAGCAGTTCAAGGGATACTGCTTCGGTTACATCTTAAAGTACAGACTACGTGCTGGTaagaa                                                                                                                                                                 >  1:507110/1‑301 (MQ=255)`  
                                                                                                                                             `gCGGTATTCTCTGCTCCAATGTCCCTTGCCACCTCTGCCCCGCAAATAACGATCAAAAGATAACCTTAGGTGAAATCCGAGCGATGGACCCACGTAAACCACATCTGAATAACCCTGAGGTAACTCCTACAGATGACCAGCCTTCCGCTGAGACAATCGAAGGTGTCACTAAGCCTTCCCACTACATGCTGTTTGACGACATTGAGGCTATCGAAGTCATTGCTCGTTCTATTACCGTTGAGCAGTTCAAGGGATACTGCTTCGGTAACCTCTTACAGTCTAGACTACGTGCCTGTaagag                                                                                                                                                                 >  1:369214/1‑300 (MQ=255)`  
                                                                                                                                             `gCGGTATTCTCTGCTCCAATGTCCCTTGCCACCTCTGCCCCGCAAATAACGATCAAAAGATAACCTTAGGTGAAATCCGAGCGATGGACCCACGTAAACCACATCTGAATAAACCTGAGGTAACTCCTACAGATGACCAGCCTTCCGCTGAGACAATCGAAGGTGTCACTAAGCCTTCCCACTACATGCTGTTTGACGACATTGAGGCTATCGAAGTGATTGCTCGTTCAATGACCGTTGAGCAGTTCAAGGGATACTGCTTCGGTAACATCTTAAAgctgtctcttatacacatctccga                                                                                                                                                                 >  1:405750/1‑278 (MQ=255)`  
                                                                                                                                                     `ctctGCTCCAATGTCCCTTGCCACCTCTGCCCCGCAAATAACGATCAAAAGATAACCTTAGGTGAAATCCGAGCGATGGACCCACGTAAACCACATCTGAATAAACCTGAGGTAACTCCTACAGATGACCAGCCTTCCGCTGAGACAATCGAAGGTGTCACTAAGCCTTCCCACTACATGCTGTTTGACGACATTGAGGCTATCGAAGTGATTGCTCGTTCAATGACCGTTGAGCAGTTCAAGGGATACTGCTTCGGTAACATCTTAAAGTACAGACTACGTGATGGTAAGAAGTCAGAGt                                                                                                                                                         <  1:518279/301‑1 (MQ=255)`  
                                                                                                                                                       `ctGCTCCAATGTCCCTTGCCACCTCTGCCCCGCAAATAACGATCAAAAGATAACCTTAGGTGAAATCCGAGCGATGGACCCACGTAAACCACATCTGAATAAACCTGAGGTAACTCCTACAGATGACCAGCCTTCCGCTGAGACAATCGAAGGTGTCACTAAGCCTTCCCACTACATGCTGTTTGACGACATTGAGGCTATCGAAGTGATTGCTCGTTCAATGACCGTTGAGCAGTTCAAGGGATACTGCTTCGGTAACATCTTAAAGTACAGACTACGTGCTGGTaagatgctgtctctt                                                                                                                                                       >  1:493899/1‑290 (MQ=255)`  
                                                                                                                                                         `gCTCCAATGTCCCTTGACAACTCTGCACCGAAAATAACGATCAAAAGATAACCTTAGGTGAAATCCGAGCGATGGACCCACGTAAACCACATCTGAATAAACCTGAGGTAACTCCTACAGATGACCAGCCTTCCGCTGAGACAATCGAAGGTGTCACTAAGCCTTCCCACTACATGCTGTTTGACGACATTGAGGCTATCGAAGTGATTGCTCGTTCAATGACCGTGGAGCAGTTCAAGGGATACTGCTTCGGTAACATCTTAAAGTACAGACTACGTGCTGGTAAGAAGTCAGAGTTAGc                                                                                                                                                     <  1:302247/301‑1 (MQ=255)`  
                                                                                                                                                          `cTCCAATGTCCCTTGCCACCTCTGCCCCGCAAATAACGATCAAAAGATAACCTTAGGTGAAATCCGAGCGATGGACCCACGTAAACCACATCTGAATAAACCTGAGGTAACTCCTACAGATGACCAGCCTTCCGCTGAGACAATCGAAGGTGTCACTAAGCCTTCCCACTACATGCTGTTTGACGACATTGAGGCTATCGAAGTGATTGCTCGTTCAATGACCGTTGAGCAGTTCAAGGGATACTGCTTCGGTAACATCTTAAAGTACAGACTACGTGCTGGTAAGAAGTCAGAGTTAGCg                                                                                                                                                    <  1:343579/301‑1 (MQ=255)`  
                                                                                                                                                            `ccAATGTCCCTTGCCACCTCTGCCCCGCAAATAACGATCAAAAGATAACCTTGGGTGAAATCCGAGCGATGGACCCACGTAAACCACATCGGAATAAACCTGAGGTAACTCCTACAGATGACCAGCCTCCCGCTGAGACAATCGAAGGTGTAACTAAGCCTTCCCACTACATGCTGTTTGACGACATTGAGGCTATCGAAGTGATTGCTCGTTCAATGACCGTTGAGCAGTTCAGGGAATACTGCTTCGGTAACATCTTAAAGTCCAGACTACGTGCTGTTAGAAAGTAAGAGTTAGCgct                                                                                                                                                  >  1:664368/1‑299 (MQ=255)`  
                                                                                                                                                               `gtataagagacagccTCTGCACCGCAAATAACGATCAAAAGATAACCTTAGGTGAAATCCGAGCGATGGACCCACGTAAACCACATCTGAATAAACCTGAGGTAACTCCTACAGATGACCAGCCTTCCGCTGAGACAATCGAAGGTGTCACTAAGCCTTCCCACTACATGCTGTTTGACGACATTGAGGCTATCGAAGTGATTGCTCGTTCAATGACCGTTGAGCAGTTCAAGGGATACTGCTTCGGTAACATCTTAAAGTACAGACTACGTGCTGGTAAGAAGTCAGAGTTAGCGTACtt                                                                                                                                               <  1:524269/288‑1 (MQ=255)`  
                                                                                                                                                                                         `aaTAACGATCAAAAGATAACCGTAGGTGAAATCCGAGCGATGGACCCACGTAAACCACATCTGAATAAACCTGAGGTAACTCCTACAGATGACCAGCCTTCCGCTGAGACAATCGAAGGTGTCACTAAGCCTTCCCACTACATGCTGTTTGACGACATTGAGGCTATCGAAGTGATTGCTCGTTCAATGACCGTTGAGCAGTTCAAGGGATACTGCTTCGGTAACATCTTAAAGTACAGACTACGTGCTGGTAAGAAGTCAGAGTTAGCGTACTTAGAGAAAGACCTAGCGAAAGCAGACt                                                                                                                     <  1:38021/301‑1 (MQ=255)`  
                                                                                                                                                                                                `aTCAAAAGATAACCTGAGGTGAAATCCGAGCGATGGACCCACGTAAACCACATCTGAATAAACCTGAGGTAACTCCTACAGATGACCAGCCTTCCGCTGAGACAATCGAAGGTGTCACTAAGCCTTCCCACTACATGCTGTTTGACGACATTGAGGCTATCGAAGTGATTGCTCGTTCAATGACCGTTGAGCAGTTCAAGGGATACTGCTTCGGTAACATCTTAAAGTACAGACTACGTGCTGGTAAGAAGTCAGAGTTAGCGTACTTAGAGAAAGACCTAGCGAAAGCAGACTTCTATaa                                                                                                              <  1:241122/301‑1 (MQ=255)`  
                                                                                                                                                                                                `aTCAAAAGAGAACCTGAGGTGAAATCCGAGCGATGGACCCACGTAAACCACATCTGAATAAACCTGAGGTAAATCCTACAGATGACCAGCCTTCCGCTGAGACAATCGAAGGGGTCACTAAGCCTTCCCACTACATGCTGTGTGACGACATTGAGGCTATCGAAGTGATTGCTCGTTCAATGACCGTTGAGCAGTTCAAGGGATACTGCTTCGGTAACATCTTAAAGTACAGACTACGTGCTGGTAAGAAGTCAGAGTTAGCGTACTTAGAGAAAGACCTAGCGAAAGCAGACTTCTATaa                                                                                                              <  1:646977/301‑1 (MQ=255)`  
                                                                                                                                                                                                   `aaaaTATAGCCTTAGGTGAAATCCGAGCGATGGACCCTCGTAAATCACATATTAGTAAACCGAAGGTAACTCCTACATATAACCATCCTTCTGATGATACAATCGAAGGTGTCACTAAGCCTTCCAACTACATGCTGGTCGACGACAAGGAAGCTATCGAAGTGATTGCTCGTTCAATGACCTTTGAGCAGTTCAAGGAATACTGCTGCGGTAAAATCTTAAAGTACTGACTACGTGCTGGTAAGAATTCAGAGTTAGCGTACTTAGAGAAAGACCTAGCGAAAGCAGACTTCTATAAAGa                                                                                                           <  1:43556/301‑1 (MQ=255)`  
                                                                                                                                                                                                    `aaaGATAACCTTAGGTGAAATCCGAGCGATGGACCCACGTAAACCACATCTGAATAAACCTGAGGTAACTCCTACAGATGTCCAGCCTTCCGCTGAGACAATCGAAGGTGTCACTAAGCCTTCCCACTACATGCTGTTTGACGACATTGAGGCTATCGAAGTGATTGCTCGTTCAATGACCGTTGAGCAGTTCAAGGGATACTGCTTCGGTAACATCTTAAAGTACAGACTACGTGCTGGTAAGAAGTCAGAGTTAGCGTACTTAGAGAAAGACCTAGCGAAAGCAGACTTCTATaactgt                                                                                                          >  1:530139/1‑297 (MQ=255)`  
                                                                                                                                                                                                    `aaaGATAACCTTAGGTGAAATCCGAGCGATGGACCCACGTAAACCACATCTGAATAAACCTGAGGTAACTCCTACAGATGACCAGCCTTCCGCTGAGACAATCGAAGGTGTCACTAAGCCTTCCCACTACATGCTGTTTGACGACATTGAGGCTATCGAAGTGATTGCTCGTTCAATGACCGTTGAGCAGTTCAAGGGATACTGCTTCGGTAACATCTTAAAGTACAGACTACGTGCTGGTAAGAAGTCAGAGTTAGCGTACTTAGAGAAAGACCTAGCGAAAGCAGACTTCTATAAAGaa                                                                                                          >  1:44872/1‑301 (MQ=255)`  
                                                                                                                                                                                                                     `aaaTACGAGCGATGGAACCACGTAAACCAAATCTGAATAAAACTGAGGTAACTCCTACAGATGACCAGCCGTCCGCTGAGACAATCGAAGGTGTCAATAAGACTTCCCACTACATGCTGTTTGACGACATTGAGGCTATCGAAGTGATTGCTCGTTCAATGACCGTTGAGCAGTTCAAGGGATACTGCTTCGGTAACATCTTAAAGTACAGACTACGTGCTGGTAAGAAGTCAGAGTTAGCGTACTTAGAGAAAGACCTAGCGAAAGCAGACTTCTATAAAGAACTCTTTGAGAAACATaa                                                                                         <  1:623542/301‑1 (MQ=255)`  
                                                                                                                                                                                                                      `aagccGAGCGATGGACCCACGTAAACCACATCTGAATAAACCTGAGGTAACTCCTACAGATGACCAGCCTTCCGCTGAGACAATCGAAGGTGTCACTAAGCCTTCCCACTACATGCTGTTTGACGACATTGAGGCTATCGAAGTGATTGCTCGTTCAATGACCGTTGAGCAGTTCAAGGGATACTGCTTCGGTAACATCTTAAAGTACAGACTACGTGCTGGTAAGAAGTCAGAGTTAGCGTACTTAGAGAAAGACCTAGCGAAAGCAGACTTCTATAAAGAACTCTTTGAGAAACAGAAg                                                                                        <  1:253457/298‑1 (MQ=255)`  
                                                                                                                                                                                                                              `cGATGGACCCACGTAAACCACATCTGAATAAACCTGAGGTAACTCCTACAGATTACCAGCCTTCCGCTGAGACAATCGAAGGTGTCACTAAGCCTTCCCACTACATGCTGTTTGACGACATTGAGGCTATCGAAGTGATTGCTCGTTCAATGACCGTTGAGCAGTTCAAGGGATACTGCTTCGGTAACATCTTAAAGTACAGACTACGTGCTGGTAAGAAGTCAGAGTTAGCGTACTTAGAGAAAGACCTAGCGAAAGCAGACTTCTATAAAGAACTCTTTGAGAAACATAAGGAtctgtc                                                                                >  1:141720/1‑296 (MQ=255)`  
                                                                                                                                                                                                                              `acagggACCCACGTAAACCACATCTGAATAAACCTGAGGTAACTCCTACAGATGACCAGCCTTCCGCTGAGACAATCGAAGGTGTCACTAAGCCTTCCCACTACATGCTGTTTGACGACATTGAGGCTATCGAAGTGATTGCTCGTTCAATGACCGTTGAGCAGTTCAAGGGATACTGCTTCGGTAACATCTTAAAGTACAGACTACGTGCTGGTAAGAAGTCAGAGTTAGCGTACTTAGAGAAAGACCTAGCGAAAGCAGACTTCTATAAAGAACTCTTTGAGAAACATAAGGATAAATg                                                                                <  1:511541/297‑1 (MQ=255)`  
                                                                                                                                                                                                                               `caTGGACCCACGTAAACCACCTCTGAATAAACCTGAGGTAACTCCTACAGATGACCAGCCGTCCGCTGAAACAATCGAAGGTGTCACTAAGCCTTCCCACTACATGCTGTTTGACGACATTGAGGCTATCGAAGTGATTGCTCGTTCAATGACCGTTGAGCAGTTCAAGGGATACTGCTGCGGTAACATCTTAAAGTACAGACTACGTGCTGGTAAGAAGTCAGAGTTAGCGTACTTAGAGAAAGACCTAGCGAAAGCAGACTTCTATAAAGAACTCTTTGAGAAACATAAGGATAAATGt                                                                               <  1:201091/300‑1 (MQ=255)`  
                                                                                                                                                                                                                                  `ggACCCACGTAAACCACATCTGAATAAACCTGAGGTAACTCCTACAGATGACCAGCCTTCCGCTGAGACAATCGAAGGTGTCACTAAGCCTTCCCACTACATGCTGTTTGACGACATTGAGGCTATCGAAGTGATTGCTCGTTCAATGACCGTTGAGCAGTTCAAGGGATACTGCTTCGGTAACATCTTAAAGTACAGACTACGTGCTGGTAAGAAGTCAGAGTTAGCGTACTTAGAGAAAGACCTAGTGAAAGCAGACTTCTATAAAGAACTCTTTGAGAAACATAAGGATAAATGTTAt                                                                            >  1:573374/1‑301 (MQ=255)`  
                                                                                                                                                                                                                                    `gacagcGTAAACCACATCTGAATAAACCTGAGGTAACTCCTACAGATGACCAGCCTTCCGCTGAGACAATCGAAGGTGTCACTAAGCCTTCCCACTACATGCTGTTTGACGACATTGAGGCTATCGAAGTGATTGCTCGTTCAATGACCGTTGAGCAGTTCAAGGGATACTGCTTCGGTAACATCTTAAAGTACAGACTACGTGCTGGTAAGAAGTCAGAGTTAGCGTACTTAGAGAAAGACCTAGCGAAAGCAGACTTCTATAAAGAACTCTTTGAGAAACATAAGGATAAATGTTATGc                                                                          <  1:363053/296‑1 (MQ=255)`  
                                                                                                                                                                                                                                    `agatgtgtataagagacaggAATAAACCTGAGGTAACTCCTACAGATGACCATCCTTCCGCTGAGACAATCGAAGGTTTCACTAAGCCTTCCCACTACATGCTGTTTGACGACATTGAGGCTATCGAAGTGATTGCTCGTTCAATGACCGTTGAGCAGTTCAAGGGATACTGCTTCGGTAACATCTTAAAGTACAGACTACGTGCTGGTAAGAAGTCAGAGTTAGCGTACTTAGAGAAAGACCTAGCGAAAGCAGACTTCTATAAAGAACTCTTTGAGAAACATAAGGATAAATGTTATGc                                                                          <  1:658118/282‑1 (MQ=255)`  
                                                                                                                                                                                                                                    `aCCCACGTAAACCACATCTGAATAAACCTGAGGTAACTCCTACAGATGACCAGCCTTCCGCTGAGACAATCGAAGGTGTCACTAAGCCTTCCCACTACATGCTGTTTGACGACATTGAGGCTATCGAAGTGATTGCTCGTTCAATGACCGTTGAGCAGTTCAAGGGATACTGCTTCGGTAACATCTTAAAGTACAGACTACGTGCTGGTAAGAAGTCAGAGTTAGCGTACTTAGAGAAAGACCTAGCGAAAGCAGACTTCTATAAAGAACTCTTTGAGAAACATAAGGATAAATGTTATGc                                                                          <  1:536001/301‑1 (MQ=255)`  
                                                                                                                                                                                                                                        `aCGTAAACCACATCTGAATAAACCTGAGGTAACTCCTACAGATGACCAGCCTTCCGCTGAGACAATCGAAGGTGTCACTAAGCCTTCCCACTACATGCTGTTTGACGACATTGAGGCTATCGAAGTGATTGCTCGTTCAATGACCGTTGAGCAGTTCAAGGGATACTGCTTCGGTAACATCTTAAAGTACAGACTACGTGCTGGTAAGAAGTCAGAGTTAGCGTACTTAGAGAAAGACCTAGCGAAAGCAGACTTCTATAAAGAACTCTTTGAGAAACATAAGGactgtctcttatacaca                                                                      >  1:159223/1‑285 (MQ=255)`  
                                                                                                                                                                                                                                        `aCGTAAACCACATCTGAATAAACCTGAGGTAACTCCTACAGATGACCAGCCTTCCGCTGAGACAATCGAAGGTGTCACTAAGCCTTCCCACTACATGCTGTTTGACGACATTGAGGCTATCGAAGTGATTGCTCGTTCAATGACCGTTGAGCAGTTCAAGGGATACTGCTTCGGTAACATCTTAAAGTACAGACTACGTGCTGGTAAGAAGTCAGAGTTAGCGTACTTAGAGAAAGACCTAGCGAAAGCAGACTTCTATAAAGAACTCTTTGAGAAACATAAGGATAAATGTTATGCAtct                                                                      >  1:566438/1‑299 (MQ=255)`  
                                                                                                                                                                                                                                        `aCGTAAACCACATCTGAATAAACCTGAGGTAACTCCTACAGATGACCAGCCTTCCGCTGAGACAATCGAAGGTGTCACTAAGCCTTCCAACTACATGCTGTTTGGCGACATTGAGGCTATCGAAGTGATTGCTCGTTCAATGACCGTTGAGAAGTTCAAGGGATACTGCTTCGGTAACATCTTAAAGTACAGACTACGTGCTGGTAAGAAGTCAGAGTTAGCGTACTTAGAGAAAGACCTAGCGAAAGCAGACTTCTATAAAGAACTCTTTGAGAAACATAAGGATACATGTTATGcctgt                                                                      >  1:308226/1‑297 (MQ=255)`  
                                                                                                                                                                                                                                             `aaCCACATCTGAATAAACCTGAGGTAACTCCTACAGATGACCAGCCTTCCGCTGAGACAATCGAAGGTGTCACTAAGCCTTCCCACTACATGCTGTTTGACGACATTGAGGCTATCGAAGTGATTGCTCGTTCAATGACCGTTGAGCAGTTCAAGGGATACTGCTTCGGTAACATCTTAAAGTACAGACTACGTGCTGGTAAGAAGTCAGAGTTAGCGTACTTAGAGAAATACCTAGCGAAAGCAGACTTCTATAAAGAACTCTTTGAGAAACATAAGGATAAATGTTATGCATAACTTCa                                                                 >  1:70171/1‑301 (MQ=255)`  
                                                                                                                                                                                                                                                 `acaTCTGAATAAACCTGAGGTAACTCCTACAGATGACCAGCCTTCCGCTGAGACAATCGAAGGTGTCACTAAGCCTTCCCACTACATGCTGTTTGACGACATTGAGGCTATCGAAGTGATTGCTCGTTCAATGACCGTTGAGCAGTTCAAGGGATACTGCTTCGGTAACATCTTAAAGTACAGACTACGTGCTGGTAAGAAGTCAGAGTTAGCGTACTTAGAGAAAGACCTAGCGAAAGCAGACTTCTATAAAGAACTCTTTGAGAAACATAAGGATAAATGTTATGCATAACTTCAAGTc                                                             <  1:3196/301‑1 (MQ=255)`  
                                                                                                                                                                                                                                                 `acaTCTGAATAAACCTGAGGTAAATCCTACAGATGACCAGCCTTCCGCTGAGACAATCGAAGGTGTCACTAAGCCTTCCCACTACATGCTGTTTGACGACATTGAGGCTATCGAAGTGATTGCTCGTTCAATGACCGTTGAGCAGTTCAAGGGATACTGCTTCGGTAACATCTTAAAGTACAGACTACGTGCTGGTAAGAAGTCAGAGTTAGCGTACTTAGAGAAAGACCTAGCGAAAGCAGACTTCTATAAAGAACTCTTTGAGAAACATAAGGATAAATGTTATGCATAACTTCAAGTc                                                             <  1:82218/301‑1 (MQ=255)`  
                                                                                                                                                                                                                                                       `gAATAAACCTGAGGTAACTCCTACAGATGACCAGCCTTCCGCTGAGACAATCGAAGGTGTCACTAAGCGTTCCCACTACATGCAGTTTGACGAAATTGAGGCTATCGAAGTGATTGCTAGTTAAATGACCGTTGAGCAGATAAAGGGATAATGCTTCGGCAACATATTAAAGTACAGACTACGTGCTGGTAAGAAGGCAGAGTGAGCGTAGTTAGAGAAAGACCTAGCGAAAGCAAACTACTATAAAGAACTATTTGAGAAACATAAGGATAAATGTTATGCATAACGTAAAGTAAAgcca                                                       >  1:163553/1‑300 (MQ=255)`  
                                                                                                                                                                                                                                                       `gAATAAACCTGAGGTAACTCCTACAGATGACCAGCCTTCCGCTGAGACAATCGAAGGTGTCACTAAGCCTTCCCACTACATGCTGTTTGACGACATTGAAGCTATCGAAGTGATTGCTCGTTCAATGACCGTTGAGCAGTTCAAGGGATACTGCTTCGGTAACATCTTAAAGTACAGACTACGTGCTGGTAAGAAGTTAGAGTTAGCGTACTTAGAGAAAGACCTAGCGAAAGCAGACTTCTATAAAGAACTCTTTGAGAAACATAAGGATAAATGTTATGCATAACTTcctgtttctaat                                                       >  1:222105/1‑290 (MQ=255)`  
                                                                                                                                                                                                                                                         `aTAAACCTGAGGTAACTCCTACAGATGACCAGCCTTCCGCTGAGACAATCGAAGGTGTCACTAAGCCTTCCCACTACATGCTGTTTGACGACATTGAGGCTATCGAAGTGATTGCTCGTTCAATGACCGTTGAGCAGTTCAAGGGATACTGCTTCGGTAACATCTTAAAGTACAGACTACGTGCTGGTAAGAAGTCAGAGTTAGCGTACTTAGAGAAAGACCTAGCGAAAGCAGACTTCTATAAAGAACTCTTTGAGAAACATAAGGATAAATGTTATGCATAACTTCAAGTCAACCCCAc                                                     <  1:331612/301‑1 (MQ=255)`  
                                                                                                                                                                                                                                                                      `aaCTCCTACAGATGACCAGCCTTCCGCTGAGACAATCGAAGGTGTCACTAAGCCTTCCCACTACATGCTGTTTGACGACATTGAGGCTATCGAAGTGATTGCTCGTTCAATGACCGTTGAGCAGTTCAAGGGATACTGCTTCGGTAACATCTTAAAGTACAGACTACGTGCTGGTAAGAAGTCAGAGTTAGCGTACTTAGAGAAAGACCTAGCGAAAGCAGACTTCTATAAAGAACTCTTTGAGAAACATAAGGATAAATGTTATGCATAACTTCAAGTCAACCCCACCTGCCGACAGccc                                        >  1:117645/1‑300 (MQ=255)`  
                                                                                                                                                                                                                                                                       `aCTCCTACAGATGACCAGCCTTCCGCTGAGACAATCGAAGGTGTCACTAAGCCTTCCCACTACATGCTGTTTGACGACATTGAGGCTATCGAAGTGATTGCTCGTTCAATGACCGTTGAGCAGTTCAAGGGATACTGCTTCGGTAACATCTTAAAGTACAGACTACGTGCTGGTAAGAAGTCAGAGTTAGCGTACTTAGAGAAAGACCTAGCGAAAGCAGACTTCTATAAAGAACTCTTTGAGAAACATAAGAATAAATGTTATGCATAACTTCAAGTCAACCCCACCTGCCGACAGCCTa                                       >  1:454842/1‑301 (MQ=255)`  
                                                                                                                                                                                                                                                                        `cTCCTACAGATGACCAGCCTTCCGCTGAGACAATCGAAGGTGTCACTAAGCCTTCCCACTACATGCTGTTTGACGACATTGAGGCTATCGAAGTGATTGCTCGTTCAATGACCGTTGAGCAGTTCAAGGGATACTGCTTCGGTAACATCTTAAAGTACAGACTACGTGATGGTAAGAAGCAAGAGTTAGTGTACTTAGAGAAAGACCTAGCGAAAGCAGACTTCTATAAAGAACTCTTTGCGAAACATAAGGATAAATGTTATGCATAAATTCAAGTCAACCCCACCTGCCGACCGCTTAt                                      >  1:284794/1‑301 (MQ=255)`  
                                                                                                                                                                                                                                                                         `cccTACAGATGACCAGCCTTCCGCTGAGACAATCGAAGGTGTCACTAAGCCTTCCCACTACATGCTGTTTGACGACATTGAAGCTATCGAAGTGATTGCTCGTTCAATGACCGTTGAGCAGTTCAAGGGATACTGCTTCGGTAACATCTTAAAGTACAGACTACGTGCTGGTAAGAAGTCAGAGTTAGCGTACTTAGAGAAAGACCTAGCGAAAGCAGACTTCTATAAAGAACTCATTGAGAAACATAAGGATAAATGTTATGCATAACTTCAAGTCAACCCCACCTGCCGACAGCCTATc                                     <  1:469613/300‑1 (MQ=255)`  
                                                                                                                                                                                                                                                                          `ccTACAGATGACCAGCCTTCCGCTGAGACAATCGAAGGTGTCACTAAGCCTTCCCACTACATGCTGTTTGACGACATTGAGGCTATCGAAGTGATTGCTCGTTCAATGACCGTTGAGCAGTTCAAGGGATACTGCTTCGGTAACATCTTAAAGTACAGACTACGTGCTTGTAAGAAGTCAGAGTTAGCGTACTTAGAGAAAGACCTAGCGAAAGCAGACTTCTATAAAGAACTCTTTGAGAAACATAAGGATAAATGTTATGCATAACTTCAAGTCAACCCCACCTGCCGACAGCCTATCt                                    >  1:629446/1‑301 (MQ=255)`  
                                                                                                                                                                                                                                                                           `cTACAGATGACCAACCTTCCTCTGAGACAATCGAAGGGGTAACTAAGCCTTCCCACCACATGCTGTTTGACGACATTGAGGCTATCGAAGTGAGTGCTCGGTCAATGACCGTTGAGCAGTTCAAGGGATACTGCTTCGGTAACATCTTAAAGTACAGACTACGTGCTGGTAAGAAGTCAGAGTTAGCGTACTTAGAGAAAGACCTAGCGAAAGCAGACTTCTATAAAGAACTCTTTGAGAAACATAAGGATAAATGTTATGCATAACTTCAAGTCAACCCCACCTGCCGACAGCCTATCtg                                   <  1:617511/301‑1 (MQ=255)`  
                                                                                                                                                                                                                                                                                    `aCCAGCCTTCCGCTGAGACAATCGAAGGTGTCACTAAGCCTTCCCACTACATGCTGTTTGACGACATTGAGGCTATCGAAGTGATTGCTCGTTCAATGACCGTTTAGCAGTTCAAGGGATCCTTCTTCGGTAACATCTTAAAGTACAGACTACGTGCTGGTAAGAAGTCAGAGTTAGCGTACTTCGAGAAAGCCCTAGCGAAAGCAGACTTCCATAAAGAACTCTTTGAGAAACATAAGGATAAATGTTATGCATCACTTCAAGTCAACCCCACCTGCCGACAGCCTATCTCATGACTTca                          >  1:542104/1‑301 (MQ=255)`  
                                                                                                                                                                                                                                                                                       `aGCCTTCCGCTGAGACAATCGAAGGTGTCACTAAGCCTTCCCACTACATGCTGTTTGACGACATTGAGGCTATCGAAGTGATTGCTCGTTCAATGACCGTTGAGCAGTTCAAGGGATACTGCTTCGGTAACATCTTAAAGTACAGACTACGTGCTGGTAAGAAGTCAGAGTTAGCGTACTTAGAGAAAGACCTAGCGAAAGCAGACTTCTATAAAGAACTCTTTGAGAAACATAAGGATAAATGTTATGCATAACTTCAAGTCAACCCCACCTGCCGACAGCCTATCTGATGAcctgtctc                       >  1:326204/1‑294 (MQ=255)`  
                                                                                                                                                                                                                                                                                        `gCCTTCCGCTGCGACAATCGAAGGTGTGACTAAGCCTTCCAACTCCATGCTGTTTGACGACCTGTAGGCTATCGAAGTGATTGCTCGTTCGATGACCGTTGAGCAGTTCAAGGGATACTGCGTAGGTAATATCTTAAAGTACAGACTACGTGCTGGTAATAAGTCAGAGTTAGCGTACTTAGAGAAAGACCTCGCGAAATCAGACTTCTATAAAGAACTCTTTGAGAAACATAAGGATAAATGTTATGCATAATTTCAAGTCAACCCCACCTGCCGACAGCCTATCTGATGACTTCACATc                      <  1:387648/301‑1 (MQ=255)`  
                                                                                                                                                                                                                                                                                        `acagtCCGCTGAGACAATCGAAGGTGTCTCTAATCCTTCCCACTACATTCTGTTTGACGACATTGAGGTTATCGGAGTGATTGCTCGTTCAATGACCGTTGAGCAGTTCCAGGGATACTTCTTCGGTAACATCTTAAAGTACAGACTACGTGCTGGTAAGAAGTCAGAGTTAGCGTACTTAGAGAAAGACCTAGCGAAAGCAGACTTCTATAAAGAACTCTTTGAGAAACATAAGGATAAATGTTATGCATAACTTCAAGTCAACCCCACCTGCCGACAGCCTATCTGATGACTTCACATc                      <  1:350459/297‑1 (MQ=255)`  
                                                                                                                                                                                                                                                                                             `ccGCTGAGACAATCGAAGGTGTCACTAAGCCTTCCCACTACATGCTGTTTGACGACATTGAGGCTATCGAAGTGATTGCTCGTTCAATGACCGTTGAGCAGTTCAAGGGATACTGCTTCGGTAACATCTTAAAGTACAGACTACGTGCTGGTAAGAAGTCAGAGTTAGCGTACTTAGAGAAAGACCTAGCGAAAGCAGACTTCTATAAAGAACTCTTTGAGAAACATAAGGATAAATGTTATGCATAACTTCAAGTCAACCCCACCTGCCGACAGCCTATCTGATGACTTCACATCTTGCt                 >  1:10540/1‑301 (MQ=255)`  
                                                                                                                                                                                                                                                                                                   `ggaCAATCGAAGGTGTCACTAAGACTTCCCACTACATGCTGTTTGACGACATTGAGGCTATCGAAGTGATTGCTCGTTCAATGACCGTTGAGCAGTTCAAGGGATACTGCTTCGGTAACATCTTAAAGTACAGACTACGTGCTGGTAAGAAGTCAGAGTTAGCGTACTTAGAGAAAGACCTAGCGAAAGCAGACTTCTATAAAGAACTCTTTGAGAAACATAAGGATAAATGTTATGCATAACTTCAAGTCAACCCCACCTGCCGACAGCCTATCTGATGACTTCACATCTTGCTCAGAgt           <  1:184760/300‑1 (MQ=255)`  
                                                                                                                                                                                                                                                                                                    `gaCAATCGAAGGTGTCACTAAGCCTTCCCACTACATGCTGTTTGACGACATTGAGGCTATCGAAGTGATTGCTCGTTCAATGACCGTTGAGCAGTTCAATGGATACTGCTTCGGTAACATCTTAAAGTACAGACTACGTGCTGGTAAGAAGTCAGAGTTAGCGTACTTAGAGAAAGACCTAGCGAAAGCAGACTTCTATAAAGAACTCTTTGAGAAACATAAGGATAAATGTTATGCATAACTTCAAGTCAACCCCACCTGCCGACAGCCTATCTGATGACTTCACATCTTGCTCAGAgtg          >  1:110954/1‑301 (MQ=255)`  

                                                                                                                                                                                                                                                                                                            `|`                                                                                                                                                                                                                                                                                                              
`GGTGTATAGCTTGCTTAGAGAAAGATGACCGTTATCCACACACCTGTAACAAAGGAGCTAACGATATGACCGAACGTGAACAAGAGATGATCATTAAGTTGATAGACAATAATGAAGGTCGCCCAGATGATTTGAATGGCTGCGGTATTCTCTGCTCCAATGTCCCTTGCCACCTCTGCCCCGCAAATAACGATCAAAAGATAACCTTAGGTGAAATCCGAGCGATGGACCCACGTAAACCACATCTGAATAAACCTGAGGTAACTCCTACAGATGACCAGCCTTCCGCTGAGACAATCGAAGGTGTCACTAAGCCTTCCCACTACATGCTGTTTGACGACATTGAGGCTATCGAAGTGATTGCTCGTTCAATGACCGTTGAGCAGTTCAAGGGATACTGCTTCGGTAACATCTTAAAGTACAGACTACGTGCTGGTAAGAAGTCAGAGTTAGCGTACTTAGAGAAAGACCTAGCGAAAGCAGACTTCTATAAAGAACTCTTTGAGAAACATAAGGATAAATGTTATGCATAACTTCAAGTCAACCCCACCTGCCGACAGCCTATCTGATGACTTCACATCTTGCTCAGAGTGGTGCCGAA  >  NC_001604.1/8224‑8824`

  

Alignment Legend

Aligned base mismatch/match (shaded by quality score): `ATCG/ATCG` `< 3 ≤` `ATCG/ATCG` `< 11 ≤` `ATCG/ATCG` `< 22 ≤` `ATCG/ATCG` `< 34 ≤` `ATCG/ATCG` `< 38 ≤` `ATCG/ATCG`

Unaligned base: `atcg`    Masked matching base: `atcg`    Alignment gap: `‑`    Deleted base: `‑`

Reads not counted as support for junction

read\_name Not counted due to insufficient overlap past the breakpoint.

read\_name Not counted due to not crossing MOB target site duplication.
